# Disparities in cardiovascular disease among Caribbean populations: a systematic literature review

**DOI:** 10.1186/s12889-015-2166-7

**Published:** 2015-08-28

**Authors:** Damian K. Francis, Nadia R. Bennett, Trevor S. Ferguson, Anselm JM Hennis, Rainford J. Wilks, E. Nigel Harris, Marlene MY MacLeish, Louis W. Sullivan, On behalf of the U.S. Caribbean Alliance for Health Disparities Research Group (USCAHDR)

**Affiliations:** Epidemiology Research Unit, Tropical Medicine Research Institute, The University of the West Indies, Kingston, West Indies Jamaica; Chronic Disease Research Centre, Tropical Medicine Research Institute, The University of the West Indies, Bridgetown, West Indies Barbados; The University of the West Indies, Kingston, West Indies Jamaica; Department of Medical Education, Morehouse School of Medicine, Atlanta, USA; The Sullivan Alliance, Alexandria, USA

## Abstract

**Background:**

Cardiovascular diseases (CVD) are the predominant cause of death globally. The large health disparities in the distribution of the burden of disease seen in developed and developing countries are of growing concern. Central to this concern is the poor outcome which is seen disproportionately in socially disadvantaged groups and racial/ethnic minorities. The aim of the study was to conduct a systematic literature review to investigate the nature of cardiovascular disease health disparities among Afro-Caribbean origin populations and identify current knowledge gaps.

**Methods:**

A systematic literature review including a detailed search strategy was developed to search MEDLINE and other research databases. Using an a priori protocol peer-reviewed publications and grey literature articles were retrieved and screened and relevant data extracted by two independent review authors. Thematic analysis was done according to CVD outcomes and measures of disparity including age, sex, ethnicity and socioeconomic status.

**Results:**

The search retrieved 665 articles of which 22 met the inclusion criteria. Most studies were conducted in the United Kingdom and centered on the prevalence of CVD by ethnicity, age and sex. An important sub-theme identified was the disparities in health service utilization/hospital admission. Coronary Heart Disease (CHD) and Peripheral Arterial Disease (PAD) were less prevalent among Afro-Caribbeans compared to Caucasian and South East Asian ethnic groups. The prevalence of CHD ranged from 0–7 % in Afro-Caribbean to 2–22 % in Caucasians. Strokes were more common among Afro-Caribbeans. There are inadequate data on morbidity and mortality from CVD, particularly across the socio-economic gradient, in Afro-Caribbean populations.

**Conclusions:**

There are important differences in morbidity and mortality from CVD across ethnic groups. Important knowledge gaps remain in understanding the social determinants of these disparities in CVD. More research exploring these gaps by varying disparity indicators needs to be undertaken.

**Electronic supplementary material:**

The online version of this article (doi:10.1186/s12889-015-2166-7) contains supplementary material, which is available to authorized users.

## Background

Cardiovascular diseases (CVD) have been established as the leading cause of morbidity and mortality in both developed and developing countries [1–3]. Low- and middle-income countries (LMICs) are disproportionally affected, with over 80 % of CVD deaths taking place in LMICs [2]. The WHO in its 2008–2013 Action Plan for the Global Strategy for the Prevention and Control of Non-communicable Diseases draws attention to the rapidity with which the increasing burden of these diseases affects poor and disadvantaged populations, contributing to widening health gaps between and within countries [4]. These gaps or “health disparities” refer to preventable differences in the indicators of health of different population groups, often defined by race, ethnicity, sex, educational level, socioeconomic status, and geographic location of residence [5].

Over the last two decades, there has been increased interest and research into health disparities, with the recognition that the unequal allocation of resources and differential access to care are a part of broader systems which may influence health in population sub-groups [6, 7]. The growing body of literature on health disparities in CVD suggests that while there has been a steady decline in CVD mortality rates in upper income countries, disparities in the burden of CVD persist, especially with regards to ethnicity, socioeconomic status and gender [8]. In countries such as the United States, health disparities continues to be pervasive and has become a growing public health concern such that its reduction and elimination is one of the main objectives of the Healthy People 2010 and 2020 campaigns [9]. Ethnic minorities such as blacks are particularly affected, with data from the United States and the United Kingdom showing higher all-cause mortality among blacks compared to whites [10–12].

Few studies have examined, the effect of social factors such as age, gender, socioeconomic status, geographic location or place of residence on differential disease patterns for cardiovascular disease prevalence and mortality in African origin populations [13, 14]. This is important because there are especially high levels of heterogeneity in cause specific mortality among these African origin sub-groups based on geographic location and country of origin [10, 14]. Additionally there is a high burden of CVD among blacks living in the Caribbean or the diaspora [15] but little is known about the social factors contributing to CVD in the region. Understanding potential differences in disease rates by ethnicity and subgroups of diverse populations is important for targeting effective interventions and policies aimed at reducing health disparities. We therefore conducted a systematic literature review in order to examine health disparities in the prevalence, incidence, and mortality rates of CVD among Afro-Caribbean populations and Caribbean immigrants compared to other ethnic groups between and within countries.

## Methods

Using the methodological framework outlined by Arskey and O’Malley [16], we examined the literature on health disparities in cardiovascular diseases limited to English-speaking Caribbean origin populations or Caribbean immigrants’ age 18 years and older. For the purposes of this review, the cardiovascular diseases were defined as coronary heart disease; cerebrovascular disease (strokes and transient ischemic attack); and peripheral arterial disease. Disparity measures included age, sex, ethnicity/race, geographic location, sexual orientation, disability status and socioeconomic status as defined by authors of included studies. Outcomes assessed included incidence and prevalence of cardiovascular disease, cardiovascular disease related mortality and health care utilization.

Studies were excluded if they did not compare cardiovascular disease morbidity or mortality within or across indicators of disparities. We also excluded studies if they reported solely on cardiovascular disease risk factors (e.g. hypertension and obesity) and those within which Caribbean populations were indistinguishably aggregated with other African origin ethnic groups. Studies not published in English were excluded due to unavailability of resources for translation.

A detailed search strategy (shown in Additional file 1) was developed to retrieve publications within major research databases and grey literature sources. The databases searched included Ovid MEDLINE, CENTRAL, LILACS, and PsycINFO. Additionally we searched Science Citation Index, Arts & Humanities Citation Index, Conference Proceedings Citation Index and Proquest. The search strategy included the key concepts ‘Caribbean region’, ‘African ancestry’ and ‘black Caribbean ethnicity’ with specific chronic diseases, and social determinants of health, health disparities, and or health inequity.

Retrieved studies were managed using EndNote X5 where duplicate articles were identified and removed from the database prior to screening. Two independent review authors screened titles and abstracts in accordance with inclusion and exclusion criteria. Discrepancies between review authors were resolved through discussion and or arbitrated by a third review author. Included studies were extracted according to a standardized study extraction form shown in Additional file 2.

Information was organized thematically according to the disparity indicators and charted using Microsoft Excel. The charting process followed a narrative approach outlined by Pawson [17], which included detailed information of population characteristics according to identified indicators of disparities. The synthesis of the charted data was conducted by a numerical analysis and thematic data charting. Numerical analysis was conducted to determine the extent, nature and distribution of the studies included in the review. The included studies were used to create tables and charts, mapping the distribution of studies according to geographic location; study design; publication year; outcome measures used to address disease entity; disparity indicators and outcome. This process subsequently informed the approach to identifying main research themes and developing an illustrative gap map. The review findings were then organized into categories which combined cardiovascular disease prevalence, incidence and mortality and disparity indicators.

## Results

### Description of included studies

**Fig. 1:**
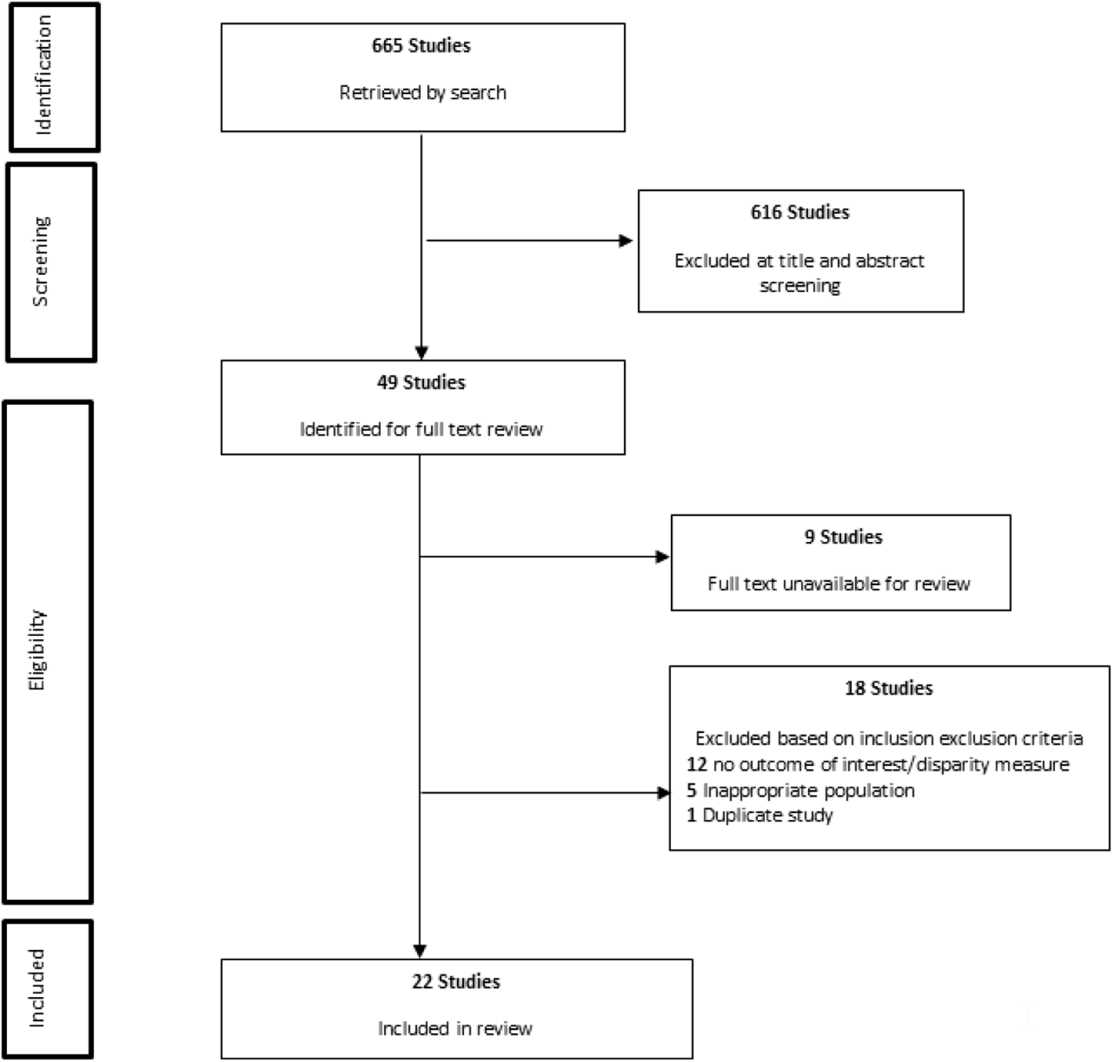
Flowchart of included and excluded papers identified for the systematic literature review

A total of 665 articles were identified from our search strategy. The process of screening and selection of included studies is outlined in a modified Preferred Reporting Items for Systematic Reviews and Meta-Analyses (PRISMA) diagram (Fig. 1). After full-text review 22 papers were included in the final synthesis.

### Characteristics of included studies

The characteristics of included studies are outlined in Additional file 3: Table S1. Fifteen of the included studies were carried out in the United Kingdom [18–32] with the remaining seven conducted in Trinidad and Tobago [33, 34], Jamaica [35–38] and the United States of America [39]. Fifteen studies were of cross sectional design; Six cohort studies [20, 26, 27, 29, 32, 33] and one case series [38]. The dates of publication ranged from 1964 to 2013 and included ten community/population based studies, ten conducted in clinic or hospital setting and the remaining two carried out in multiple settings (See Additional file 3: Table S1). The populations of comparison included Afro-Caribbean, Indo-Caribbean, South-Asians, Caucasians, Chinese, African American and African Blacks. The cardiovascular disease outcomes reported on in the included studies were: coronary heart disease; peripheral arterial disease; cerebrovascular disease; and overall CVD.

### Themes and disparity indicators

The prevalence of CVD was the most common theme, being reported in 12 of the 22 studies [18, 19, 21–26, 29, 31, 35, 36]. Ten studies reported on mortality [20, 27–30, 32, 34, 37–39] and three reported on incidence of CVD [27, 32, 33]. Only a few sub-themes were identified with three studies reporting the prevalence of smoking, three studies included health service utilization/hospital admission [23, 24, 26] and obesity [18, 19], while a single study reported on the prevalence of the metabolic syndrome [21].

### Disparities in prevalence of CVD

Overall, the prevalence of coronary heart disease and peripheral artery disease is lower in Afro-Caribbean populations [19, 21, 24–26, 29, 31] when compared to other ethnic groups except for Immigrant Chinese and African Blacks to the UK [19, 29]. Prevalence of CHD ranged from 0–7 % [21, 31] in Afro-Caribbean compared to 2–22 % [21, 31] in Caucasians. Only two studies [23, 31] reported no difference in the prevalence of CVD among Afro-Caribbean compared to other ethnic groups. Two studies reported a higher prevalence of cerebrovascular disease among Afro-Caribbeans, Caucasians and Indo-Asians [22, 24]. Wang et al. [29] reported that Afro-Caribbeans have similar prevalence to African Blacks and Whites in South London. Whereas Gill et al. [25] reported a higher prevalence of stroke/TIA among South Asians compared to Afro-Caribbeans enrolled in the Ethnic-Echocardiographic Heart of England Screening Study. The prevalence of overall CVD in Afro-Caribbean was not different from other ethnic groups [18, 22, 23] in three out of six studies. Few studies reported on differences in CVD morbidity and mortality by age and socioeconomic status among other measures of disparities.

The prevalence of coronary heart disease was lower among Afro-Caribbean men compared to all ethnic group except Chinese (Afro-Caribbean 0.8 %; Black African 1.6 %; South Asians 2.5 %; Caucasian 2.4 %; and Chinese 0.9 %) [19]. Within the Afro-Caribbean ethnic group the prevalence of acute coronary syndrome is higher in women compared to men [35]. Ferguson [35] also reported that CVD increased with age in the Afro-Caribbean population.

### Disparities in the incidence of CVD

The incidence of coronary heart disease was lower in Afro-Caribbeans compared to Indians in Trinidad and Tobago, Caucasians and South Asians [27, 32, 33]. In particular, Khattar et al. [27] found that the incidence of coronary heart disease was significantly higher among Whites (1.32 per 100 person years) and South Asians (2.86 per 100 person years) compared to Afro-Caribbeans (0.18 per 100 person years) [27]. Incidence of coronary heart disease increased with age but only in men [32]. In sex specific analysis Miller reported a higher incidence of coronary heart disease among men compared to women. Differences seen across ethnicity by sex was not statistically significant but of note it was higher in Afro-Caribbean women compared to European counterparts [33] and one study reported no sex difference in the incidence of CVD [33]. The UKPDS trial reported that Afro-Caribbean men and women had lower socioeconomic status compared to Caucasians and South Asians living in the UK but provided no report on outcome by this disparity indicator [32].

### Cardiovascular disease mortality

Our systematic literature review found that coronary heart disease mortality is significantly lower among Afro-Caribbean compared to South Asian, Caucasian, US Blacks and East African ethnic groups [20, 27, 30, 32, 39]. According to Chaturvedi et al. [20], when compared to Caucasians, Afro-Caribbeans had a significantly lower sex adjusted mortality rate from ischaemic heart disease HR 0.40 (0.17, 0.92). This difference remained even after adjustment for smoking, though not statistically significant [HR 0.64 (0.26, 1.58)] [20] and was a recurring sub-theme in our review of disparities in CVD among Afro-Caribbean Populations [18–20].

Place of birth also emerged as a disparity theme in our analyses. In the single study conducted in the United States in this review [39], Southern born US Blacks had a standardized rate of death from coronary heart disease more than twice that of Caribbean-born Blacks (406.5 vs. 165.2 per 100,000) in the less than 65 years age group [39]. Coronary heart disease deaths were also lower in Caribbean-born Blacks compared to US Northeastern Whites. On the other hand in all age groups and both sexes, deaths due to stroke were higher among Caribbean-born blacks than among US Northeastern-born Whites [39].

Three studies found that coronary heart disease mortality was higher among men compared to women [30, 32, 38]. A single study conducted in a mixed ethnic Caribbean population found that 5 year mortality rate from strokes was lower in women compared to men (Incidence 134 vs. 185/100,00 per year) and was in higher in the 35–74 years old group compared to those 75–84 years for both men and women [34]. One study also reported that Caribbean-born black men had lower rates of death from coronary heart disease than US Northeastern-born whites in all age groups [39].

The published literature is less clear on ethnic differences in stroke mortality [27–29]. Two studies showed no differences in mortality rates across ethnic groups [27, 29], and one found that stroke mortality was higher in the Afro-Caribbean population of Barbados when compared to mortality among mixed Caucasian and Afro-Caribbean immigrants to the UK in the South London Stroke Registry (SLSR) [28]. One important limitation of the SLSR was that although it included a 15 % Afro-Caribbean group, it was not disaggregated in the analysis so a direct comparison with the Barbados population could not be made.

### Prevalence of peripheral arterial disease

Prevalence of peripheral arterial disease was lower in Afro-Caribbeans when compared to Caucasians [21] but higher than that of South Asians [21, 25]. Ferguson and colleagues reported no significant gender differences in peripheral arterial disease in diabetic persons in Jamaica [35].

### Health care utilization and access

Two studies reported health care access through admission rates across ethnic groups and found no difference in access to care for ischemic heart disease, heart attack or stroke by sex [23, 24]. Goyal and colleagues also reported no evidence of differences in patterns of referral and or treatment by ethnicity [26].

## Discussion

Our systematic literature review examined and synthesized the existing literature on disparities in cardiovascular disease morbidity and mortality among Caribbean origin populations. The study used a predefined protocol with a systematic approach to identify, retrieve and screen relevant studies for inclusion. Overall this review presents a summary of health disparities in cardiovascular disease prevalence, incidence and mortality as well as emerging themes such as health care utilization and access.

**Fig. 2:**
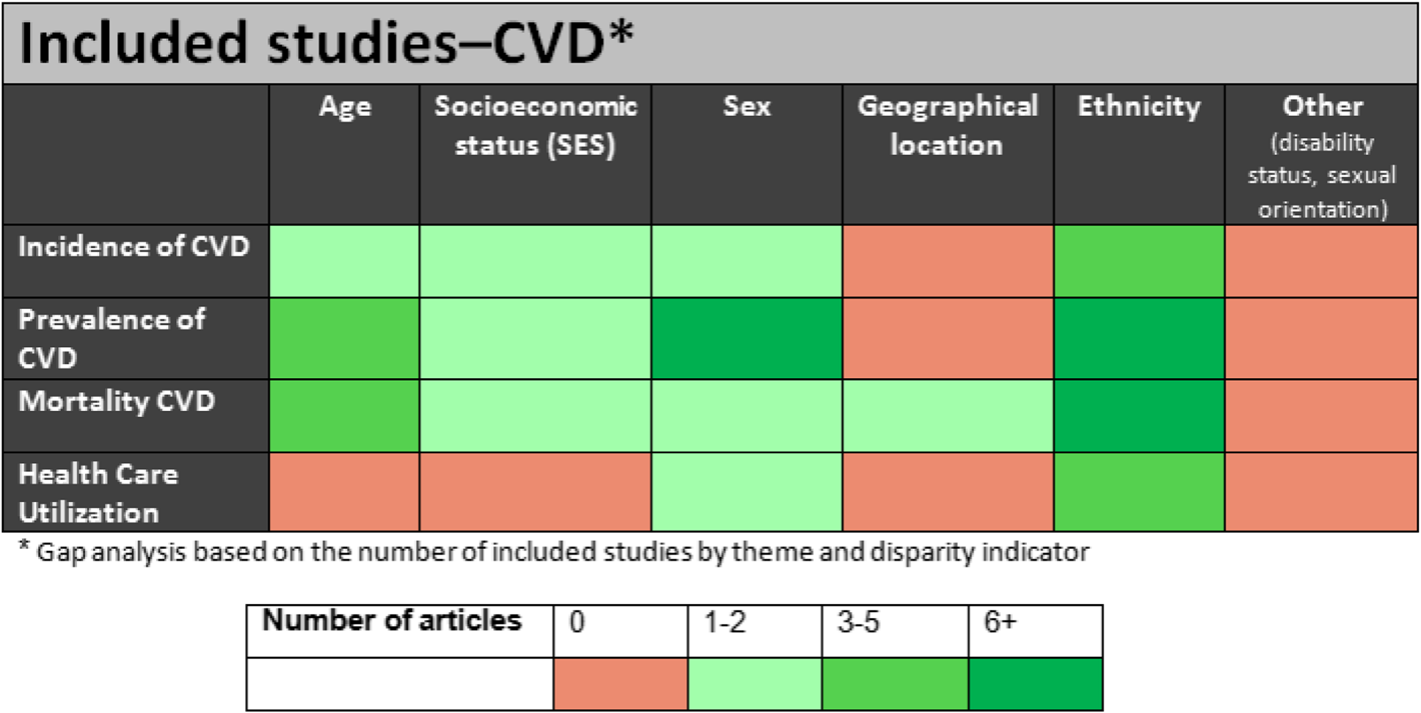
Gaps in health disparities research literature according to outcomes and disparity indicators

There remain significant knowledge gaps within the CVD literature with very few studies reporting on socioeconomic status and geographic location. There were no studies which reported on CVD by sexual orientation or in the disabled population (Fig. 2). Limited data was also reported for the health utilization and access theme.

In this review we found that: most of the studies were conducted in the United Kingdom and used a cross sectional design; prevalence and mortality of cardiovascular disease were the most reported outcome and was often stratified by ethnicity, sex and age; the prevalence of coronary heart disease and peripheral artery disease was lower in Afro-Caribbean populations compared to Caucasians and South Asians; the incidence of CHD was lower among Afro-Caribbeans compared to other ethnic groups. Cerebrovascular disease prevalence is higher among Afro-Caribbeans when compared to Caucasians and South Asians but similar to other blacks. Whilst CHD mortality is lower, stroke mortality is higher in Afro-Caribbeans compared to other ethnic groups.

Our findings on the prevalence and incidence of coronary heart disease and cerebrovascular disease supports previous reports by Yusuf and Colleagues [1] as well as contributes new information to what is known when comparing Caribbean Blacks to global populations of different ethnicity. The seminal publication on global burden of cardiovascular disease found, that coronary heart disease rates were higher among South Asians compared to other ethnic groups [1]. Our systematic literature review reports a lower burden of coronary heart disease in Afro-Caribbeans compared to South Asians and Whites.

Earlier reviews [1, 40] and data from the US Census Bureau [41], support our findings that cerebrovascular disease morbidity and mortality remains higher among African origin populations including Afro-Caribbeans. Davis et al. [40] conducted a systematic review which examined cardiovascular health disparities and found the prevalence of CHD to be lower among black men but the opposite in women. Our findings are largely consistent with these reports but it must be noted that this systematic literature review examined studies comparing Afro-Caribbean populations to other ethnic groups whereas the cited reviews used the African American as a proxy for African origin populations.

Although research in health disparities has received priority over the last several years [6, 7], our review has highlighted gaps in well-established markers such as socio-economic status. Socio-economic status, such as income and education, has been consistently reported as an important social determinant of health [42, 43]. More recently it is being explored as a modifiable factor to reduce the disparities seen in cardiovascular disease and its risk factors [44]. This review found very few studies investigating socio-economic status and CVD among Afro-Caribbeans. The gap was even wider in studies exploring less talked about markers such as geographic location, disability status and sexual orientation. These indicators have recently received some attention and are now being reported as part of the CDC health disparities and inequalities report [45].

One limitation of this review was that nine studies were inaccessible due to historic age of publication. Another limitation was the restriction of the review to studies published in English language which may have resulted in missing potentially relevant studies.

## Conclusions

Our systematic literature review found that differences in CVD burden and mortality exist between Afro-Caribbeans and other ethnic groups including African Americans living in similar and different geographic locations. The literature gap on CVD burden by ethnicity has improved over the last 10 years but there is still a dearth of information on other determinants such as disability status and sexual orientation. Differences in health care access and utilization was noted to be an emerging theme but there is little published data on differences in Afro-Caribbeans compared to other ethnic groups. It is important that researchers in health disparities pay attention to indicators such as socio-economic status, geographic location and appropriate definition and classification of ethnic groups to allow for tailored interventions aimed at decreasing health disparities.

## Additional files

Additional file 1:
**Search strategy.** A detailed description of the search strategy and databases searched to identify relevant papers for inclusion in this systematic literature review. (DOCX 21 kb) Additional file 2:
**Data extraction domains.** (DOCX 21 kb) Additional file 3:
**Table outlining the components of the data extraction form to which information was abstracted from included studies for this systematic literature review.** (DOCX 24 kb)
