# Supplementary material for: Disparities in cardiovascular disease among Caribbean populations: a systematic literature review
Source: BMC Public Health. 2015 Aug 28;15:828. doi: 10.1186/s12889-015-2166-7 (PMC4551768; doi:10.1186/s12889-015-2166-7)
Supplement: Additional file 1: — Search strategy. A detailed description of the search strategy and databases searched to identify relevant papers for inclusion in this systematic literature review. (DOCX 21 kb) [file 12889_2015_2166_MOESM1_ESM.docx]

***Additional file 1: Search Strategy***

Detailed search strategy used to identify relevant papers for this scoping review.

Database: Ovid MEDLINE(R) In-Process & Other Non-Indexed Citations and Ovid MEDLINE(R) <1946 to Present>

Search Strategy:

--------------------------------------------------------------------------------

1 Caribbean/eh (575)

2 African Americans/eh, ge (3174)

3 Caribbean Region/eh (575)

4 African Continental Ancestry Group/eh, mo, ed, sn (2998)

5 Lucayan Archipelago/ (0)

6 Bahamas/ (309)

7 Turks.mp. and Caicos Islands/ [mp=title, abstract, original title, name of substance word, subject heading word, keyword heading word, protocol supplementary concept, rare disease supplementary concept, unique identifier] (0)

8 Turks Island/ (0)

9 Caicos Island/ (0)

10 Greater Antilles/ (0)

11 Cuba/ (4051)

12 Hispaniola/ (0)

13 Haiti/ (2172)

14 Dominican Republic/ (1079)

15 Jamaica/ (2848)

16 Cayman Islands/ (3153)

17 Puerto Rico/ (4798)

18 Lesser Antilles/ (0)

19 Leeward Islands/ (0)

20 United States Virgin Islands/ (180)

21 US Virgin Islands/ (0)

22 Saint Croix/ (0)

23 Saint Thomas.mp. (39)

24 Saint John/ (0)

25 Water Island/ (0)

26 British Virgin Islands/ (3153)

27 Tortola/ (0)

28 Virgin Gorda/ (0)

29 Anegada/ (0)

30 Jost Van Dyke/ (0)

31 Anguilla/ (1289)

32 Antigua/ (82)

33 Barbuda/ (82)

34 Redonda/ (0)

35 Saint Martin/ (0)

36 Sint Maarten/ (0)

37 Saba/ (0)

38 Sint Eustatius/ (0)

39 Saint Barthelemy/ (0)

40 Saint Barth*lemy/ (0)

41 Saint Kitts/ (44)

42 Nevis/ (44)

43 Montserrat/ (3153)

44 Guadeloupe/ (292)

45 French Antilles/ (0)

46 Les Saintes/ (0)

47 Marie*Galante/ (0)

48 La Desirade/ (0)

49 La D*sirade/ (0)

50 Windward Islands/ (0)

51 Dominica/ (64)

52 Martinique/ (366)

53 Saint Lucia/ (45)

54 St Lucia/ (45)

55 "Saint Kitts and Nevis"/ (44)

56 Saint Vincent/ (35)

57 St Vincent/ (35)

58 Grenadines/ (35)

59 Grenada/ (77)

60 Carriacou/ (0)

61 Petite Martinique/ (0)

62 Barbados/ (499)

63 Trinidad/ (1370)

64 Tobago/ (1370)

65 Leeward Antilles/ (0)

66 Aruba/ (3153)

67 Curacao/ (0)

68 Bonaire/ (0)

69 West Indies/ (3153)

70 West Indian/ (0)

71 West India/ (0)

72 West Indi*/ (0)

73 St thomas.mp. (1261)

74 St John.mp. (296)

75 St Martin.mp. (49)

76 st Maarten.mp. (13)

77 Netherlands Antilles/ or st Eustatius.mp. (256)

78 st Barthelemy.mp. (12)

79 st Kitts.mp. or "Saint Kitts and Nevis"/ (124)

80 "Virgin Islands of the United States"/ or St Croix.mp. (278)

81 Guyana/ or Guyana$.tw. (837)

82 Suriname/ or Surinam$.tw. (1182)

83 (French adj Guyana).mp. (184)

84 Belize/ or Belize.mp. (618)

85 anguilla$.tw. (2680)

86 (antigua adj2 barbuda).tw. (32)

87 antigua$.tw. (160)

88 barbud$.tw. (41)

89 aruba$.tw. (90)

90 bonaire.tw. (28)

91 curacao$.tw. (247)

92 baham$.tw. (550)

93 barbad$.tw. (1089)

94 beliz$.tw. (512)

95 bermud$.tw. (1052)

96 tortola$.tw. (28)

97 curacao.tw. (244)

98 (virgin adj2 gorda).tw. (0)

99 anegad$.tw. (4)

100 cayman$.tw. (166)

101 cuba$.tw. (5421)

102 dominica$.tw. (1863)

103 grenad$.tw. (430)

104 grenad$.tw. (430)

105 haiti$.tw. (2521)

106 jamaica$.tw. (3308)

107 montserrat$.tw. (86)

108 (puerto adj2 rico).tw. (4160)

109 martiniqu$.tw. (526)

110 guadelop$.tw. (4)

111 saint kitts-nevis.tw. (1)

112 (nevis$ or kitt$).tw. (3904)

113 lucia$.tw. (573)

114 eustatius.tw. (8)

115 (maarten$ or martin$).tw. (6867)

116 barthelem$.tw. (56)

117 saba.tw. (308)

118 trinidad$.tw. (1793)

119 tobago$.tw. (596)

120 (virgin adj2 island$).tw. (357)

121 USVI.tw. (34)

122 (thomas$ or croix$ or john$).tw. (98780)

123 (west adj2 indi$).tw. (3534)

124 caribbean$.tw. (8317)

125 leeward.tw. (117)

126 antill$.tw. (781)

127 windward.tw. (103)

128 guyan$.tw. (1019)

129 surinam$.tw. (1048)

130 guian$.tw. (1304)

131 hispaniol$.tw. (151)

132 doming$.tw. (599)

133 or/1-132 (160218)

134 *Cardiovascular Diseases/ (63802)

135 *Heart Diseases/ (38016)

136 *Diabetes Mellitus/ (60718)

137 diabetes.mp. (395222)

138 Hypertension/ (188116)

139 (high adj blood adj pressure).mp. (10187)

140 Stroke/ (54028)

141 Pulmonary Disease, Chronic Obstructive/ (21609)

142 Bronchitis, Chronic/ (783)

143 (chronic adj2 obstructive adj2 pulmonary).tw. (27285)

144 (chronic adj2 obstructive adj2 airway$).tw. (833)

145 (chronic adj2 bronchitis).tw. (9847)

146 copd.mp. (24626)

147 Emphysema.mp. (27395)

148 Emphysema/ (4570)

149 cancer.tw. (998563)

150 neoplasm$.tw. (96179)

151 exp neoplasms/ or exp leukemia/ or exp lymphoma/ or exp lymphoma, non-hodgkin/ or exp "neoplasms, connective and soft tissue"/ or exp abdominal neoplasms/ or exp anal gland neoplasms/ or exp bone neoplasms/ or exp breast neoplasms/ or exp digestive system neoplasms/ or exp endocrine gland neoplasms/ or exp eye neoplasms/ or exp "head and neck neoplasms"/ or exp hematologic neoplasms/ or exp mammary neoplasms, animal/ or exp nervous system neoplasms/ or exp pelvic neoplasms/ or exp skin neoplasms/ or exp soft tissue neoplasms/ or exp splenic neoplasms/ or exp thoracic neoplasms/ or exp urogenital neoplasms/ (2507280)

152 Depression/ (72366)

153 mental health/ or exp mental disorders/ or exp adjustment disorders/ or exp anxiety disorders/ or exp delirium, dementia, amnestic, cognitive disorders/ or exp dissociative disorders/ or exp eating disorders/ or exp factitious disorders/ or exp impulse control disorders/ or exp mental disorders diagnosed in childhood/ or exp mood disorders/ or exp neurotic disorders/ or exp personality disorders/ or exp "schizophrenia and disorders with psychotic features"/ or exp "sexual and gender disorders"/ or exp sleep disorders/ or exp somatoform disorders/ or exp substance-related disorders/ (1104891)

154 exp Asthma/ (105350)

155 asthma.mp. (134511)

156 or/134-155 (4621636)

157 Socioeconomic Factors/ (109574)

158 Health Status Disparities/ (6870)

159 (social adj2 determinants adj2 health).mp. (1045)

160 (gender or sex).mp. [mp=title, abstract, original title, name of substance word, subject heading word, keyword heading word, protocol supplementary concept, rare disease supplementary concept, unique identifier] (695238)

161 Ethnic Groups/ or ethnicity.mp. (69773)

162 Age Factors/ (364634)

163 Educational Status/ (38804)

164 Income/ (20840)

165 Occupations/ (16755)

166 socioeconomic.mp. (141152)

167 sexual orientation.mp. (2566)

168 (gay or homosexual or lesbian).mp. [mp=title, abstract, original title, name of substance word, subject heading word, keyword heading word, protocol supplementary concept, rare disease supplementary concept, unique identifier] (14244)

169 Poverty Areas/ or Poverty/ (29695)

170 Urban Population/ or Urban Health/ (60795)

171 Rural Health/ or Rural Population/ or Rural Health Services/ (66001)

172 or/157-171 (1223690)

173 133 and 156 and 172 (6881)

174 limit 173 to yr="2013" (166)

175 or/85-132 (147358)

176 175 and 156 and 172 (5081)

177 limit 176 to yr="1860 - 2012" (4942)
