# Supplementary material for: Disparities in cardiovascular disease among Caribbean populations: a systematic literature review
Source: BMC Public Health. 2015 Aug 28;15:828. doi: 10.1186/s12889-015-2166-7 (PMC4551768; doi:10.1186/s12889-015-2166-7)
Supplement: Additional file 2: — Data extraction domains. (DOCX 21 kb) [file 12889_2015_2166_MOESM2_ESM.docx]

**Additional file 2: Data Extraction Domains**

The table shows details of the data extraction form used to abstract information for the systematic literature review.

| **Field** | **Field Description and Instructions** | **Allowable Values** |
| --- | --- | --- |
| Study ID | Insert any of the following here: PMID (accession number) , doi, ISSN, ISBN | alphanumeric |
| First Author | Enter surname in lower case at all times using the following format. [surname][,][initial 1][initial2] | text |
| Publication year | Enter the year that the study was published from the drop down menu | text |
| Title | Enter the exact title of the paper as it appears in the journal | text |
| Study location | Enter the country or countries, in which the study was carried out | text |
| Study aims/objectives | Enter the objectives of the paper and after the entry, it should be stated if the objectives entered were [stated][inferred]or [not known] | text |
| **METHODOLOGY** | | |
| Study Design | Classify the study broadly as randomized or non-randomized studies. Non-randomized studies should be classified as 1. Controlled before-and-after-study 2. Interrupted-time-series study, 3. Historically controlled study, 4. Cohort study, 5. Case-control study 6. Cross-sectional study, 7. Case series (uncontrolled longitudinal study). 8. Other (indeterminate). For non-randomized studies that do not report specific study design two review authors will assess the study design. | text |
| Description | Enter population based, hospital-based, community-based from drop-down menu | text |
| study began | Enter information provided by the paper in the following (when all fields are available) [date]-[month]-[year] | text |
| study ended | Enter information provided by the paper in the following (when all fields are available) [date]-[month]-[year] | text |
| Selection | Enter the inclusion and exclusion criteria given by the study | text |
| Predictors | Enter the disease the paper addressed. E.g. Diabetes, lung cancer etc. | text |
| outcome | Enter the outcome that the paper addressed e.g. mortality or health care utilization | text |
| outcome measures | Enter the outcome measure that was used in the study e.g. odds ration or relative risk | text |
| disparity measures | Enter the disparity variable that was used in the paper e.g. socioeconomic status which should not be abbreviated, and if more specific variables e.g. education were used they should all be specified | text |
| **Population characteristics** | This section describes the baseline characteristics of the study population (frequency distribution/proportions). Where there are no information for subsections within this category the word [not stated] should be used. | |
| age range | Enter the age range of study participants | text |
| sex/gender distribution | Enter the total number of participants as well as the number and percentage of participants that are male in the following format *n (%)* | text |
| ethnicity/race | Enter the total number and percentage of participants by ethnicities that were defined in the studies (including non- afro Caribbean) in the following format *n*(%) | text |
| sexual orientation | Enter the total number and percentage of participants by sexual orientation that were defined in the studies (homosexual, hetero-sexual, trans-gendered, other/not specified) in the following format *n*(%) | text |
| disability status | enter the total number and percentage of the participants who were disabled n(%) (state definition of disability if present in manuscripts) | text |
| socioeconomic status | Enter which socioeconomic variable(s) was reported by the study as well as the number and percentage that fell into each sub-category (e.g. Education, Occupation, Income, etc.) | text |
| **MAIN FINDINGS** | If no information was specified in the next 5 sub-sections the word [not stated] should be used. Report both univariate (UV) and multivariate findings (MV). If multivariate state the other variables in the model | |
| age | Enter the results or findings relating to outcome or disparity measured reported/stratified by age group/range. | text |
| sex | Enter the results or findings relating to outcome or disparity measured reported/stratified by sex/gender (male vs female). | text |
| ethnicity/race | Enter the results or findings relating to outcome or disparity measured reported/stratified by ethnicity/race | text |
| location | Enter the results or findings relating to outcome or disparity measured reported/stratified by different area of residence (urban vs rural) | text |
| sexual orientation | Enter the results or findings relating to outcome or disparity measured reported/stratified by sexual orientation (homosexual, hetero-sexual, trans-gendered, and other/not specified). | text |
| disability status | Enter the results or findings relating to outcome or disparity measured/ stratified by disability as it was stated by the paper | text |
| socioeconomic status | Enter the results or findings relating to outcome or disparity measured reported/stratified by socioeconomic status composite or single components. | text |
| CONCLUSION | Enter the conclusion that was stated by the authors in the paper, if no conclusion was stated indicate this by [not stated] | text |
| LIMITATIONS STATED | Enter the limitations that were stated by the authors of the paper | text |
| OVERALL CONCLUSION ON STUDY | Enter reviewer overall conclusion on study based on authors conclusion and limitations of the study (stated or unstated) | text |
| SCOPING REVIEW CLASSIFICATION 1 | Click the drop down menu and select the option that best categorizes the paper | text |
| SCOPING REVIEW CLASSIFICATION 2 | Click the drop down menu and if a second category defines the paper select it | text |
| INCLUDE/EXCLUDE | Enter the word [include] or [exclude], to indicate whether or not the paper should be included in the scoping review | text |
| REVIEWED BY | Enter the initials of the review author that extracted the data | text |
