# Supplementary material for: Disparities in cardiovascular disease among Caribbean populations: a systematic literature review
Source: BMC Public Health. 2015 Aug 28;15:828. doi: 10.1186/s12889-015-2166-7 (PMC4551768; doi:10.1186/s12889-015-2166-7)
Supplement: Additional file 3: — Table outlining the components of the data extraction form to which information was abstracted from included studies for this systematic literature review. (DOCX 24 kb) [file 12889_2015_2166_MOESM3_ESM.docx]

***Additional file 3: Table S1: Summary of CVD included articles***

| **Author**  **(Year)** | **Title** | **Country(s)** | **Setting(Hospital/**  **Clinic/**  **Community )** | **Study Design** | **Population** | **Themes** | **Sub Themes** | **Disparity Measure** | **Quality/Limitations** |
| --- | --- | --- | --- | --- | --- | --- | --- | --- | --- |
| Abbots, J 2004 | Cardiovascular risk profiles in UK-born Caribbeans and Irish living in England and Wales | United Kingdom | Community | Cross sectional | Caribbean n= 206, UK born Irish n= 453, UK born general population n= 2417,  Age range: 35-54 years | Prevalence of CVD risk factors | Smoking  Obesity | Age, Sex, Ethnicity, Social capital | No reported limitations. Inadequate sample size of the Caribbean population to facilitate subgroup analysis. |
| Brindle, P 2006 | Primary prevention of cardiovascular disease: a web-based risk score for seven British black and minority ethnic groups | United Kingdom | Community | Cross Sectional | Ethnicity: Men- South Asians n= 617, Chinese n= 132, Afro-Caribbean n= 137, African Blacks n=41, Irish 247  Women- South Asians n= 610, Chinese n= 195, Afro-Caribbean n= 284, African Blacks n=87, Irish 312  Age range: 35-54 yrs. | Prevalence of CHD | Smoking and obesity | Ethnicity, Sex | No reported limitations. |
| Chaturvedi, N 1996 | Differences in mortality and morbidity in African Caribbean and European people with non-insulin dependent diabetes mellitus: results of 20 year follow up of a London cohort of a multinational study | United Kingdom | Hospital &  Community | Cohort study | Afro-Caribbean n= 77, European n= 150  Mean age :  European and Afro-Caribbean Women 48 yrs.  European men 48yrs, Afro Caribbean men 47 yrs. | Mortality from IHD , All-cause mortality | smoking | Sex, Ethnicity | The numbers of persons, particularly of African Caribbeans, are small and cannot support full analyses separately by sex. Relevant confounders were not collected for adjustment of mortality data. |
| Chaturvedi, N  1994 | Relationship of glucose intolerance to coronary risk in Afro-Caribbeans compared with Europeans | United Kingdom | Clinic | Cross sectional | Caucasians n= 50, mean age 61.6 + 1.3 yrs.  Asians n= 30, mean age 56.3 + 2.0 yrs.  Afro-Caribbean n= 18, mean age 58.3 + 1.9 yrs. | Prevalence of IHD & PVD | Metabolic syndrome | Ethnicity | No reported limitations. Prevalence rates are expressed after directly standardizing to the combined age distribution of the study population. |
| Conway, D 2004 | Comparison of outcomes of patients with symptomatic peripheral artery disease with and without atrial fibrillation (the West Birmingham Atrial Fibrillation Project) | United Kingdom | Hospital | Cross sectional | Indo-Asian n= 23, mean age 64 + 11yrs  Afro-Caribbean n= 44, mean age 72 + 8yrs  Caucasian n= 320, mean age 70 + 12  Gender: 59% men | Prevalence of IHD | Emergency admission | Ethnicity | No reported limitations. |
| Conway, D 2003 | Ethnicity in Relation to Atrial Fibrillation and Stroke (the West Birmingham Stroke Project) | United Kingdom | Hospital | Cross sectional | Caucasians n= 582  Indo-Asian n= 116  Afro-Caribbean n= 134  Mean age 74+ 12yrs | Prevalence of prior stroke, Odds of stroke attributed to DM | Death during hospitalization | Ethnicity, Sex, Age | No reported limitations. |
| Cruickshank, J 1980 | Heart attack, stroke, diabetes, and hypertension in West Indians, Asians, and whites in Birmingham, England | United Kingdom | Hospital | Cross sectional | Men 15624,  Europe/UK n= 11157(71.4%)  West indies n= 1227(7.9%) Pakistani n= 2289(14.7)  Unknown n= 951(6.1%)  Women 12043  Europe/UK n= 9232(76.7%)  West indies n= 1048(8.7%) Pakistani n= 1156(9.6%)  Unknown n= 604(5.0%) | Frequency of Heart attack and Strokes | Hospital admission | Ethnicity, Sex | No reported limitations. Short paper, letter to the editor. |
| Fang, J  1996 | The association between birthplace and mortality from cardiovascular causes among black and white residents of New York City | United States of America | Community | Cross sectional | US Blacks south n=366,853, US Black North east n=1,008,677, Afro- Caribbean n=309,380 [16.6%])Whites in the north east 2,315,288  Age: US Blacks south 51.1±17.2 US Black North east 23.2±17.7 Afro-Caribbean 37.7±17.9 Whites in the north east 39.6±23.1 | Prevalence of CVD |  | Ethnicity | Age adjusted mortality rates with standardized mortality ratios. Undercounting of migrants in the census data may have increased the mortality rates of the Caribbean-born and thus would tend only to diminish, not heighten, any observed differences in mortality. |
| Ferguson, T 2010 | Cardiovascular disease among diabetic in-patients at a tertiary hospital in Jamaica | Jamaica | Hospital | Cross sectional | Men n=288  Women n=516  Age range 18-105 yrs. | Prevalence of IHD, Acute Coronary Syndrome (ACS), PVD & Stroke |  | Age, Sex | No reported limitations. Short paper, letter to the editor. |
| Fodor, T  1964 | Myocardial Disease in a Rural Population in Jamaica | Jamaica | Community Health Centre | Cross sectional | Men n= 300  Women n= 300  Age range 35-64yrs | Prevalence of MI |  | Age, Sex, | No reported limitations. |
| Gill, P  2011 | Prevalence of Heart Failure and Atrial Fibrillation in Minority Ethnic Subjects: The Ethnic-Echocardiographic Heart of England Screening Study (E-ECHOES) | United kingdom | Community health centre | Cross sectional | South Asians n= 3442  Afro-Caribbean n= 1912  Median age 58 Inter-Quartile Range (51,70)  Gender 52.5% female | Prevalence of MI, PAD, Stroke | Deprivation index | Ethnicity | large sample of under-represented  hard to reach groups drawn from 20 centres increasing  generalizability to these minority ethnic groups. Response rate was low (49.6%). |
| Goyal, D  2007 | South Asian or Afro-Caribbean ethnicity is not associated with altered 1min heart rate recovery estimates in suspected coronary artery disease patients | United Kingdom | Clinic | Cohort study | Caucasians n= 159  South Asians n= 80  Caribbean n= 32  Age range 40-75 yrs. | Prevalence of CVD | Health care utilization | Ethnicity | No reported limitations. |
| Khattar, R 2000 | Racial variation in cardiovascular morbidity and mortality in essential hypertension | United Kingdom | Hospital & Community | Cohort study | White n= 528 (male 62%)  South Asian n= 106 (male 79%)  Afro-Caribbean n=54 (male 54%)  Mean age: White 52.2(10.9)  South Asian 46.3(9.0)  Afro-Caribbean 46.8 (9.1) | Mortality, Incidence |  | Ethnicity | Inherent biases of the retrospective manner in which data on some measures were collected. Varying time lag between baseline and follow-up clinic visit increases information bias. |
| Miller, G 1989 | Ethnicity and other characteristics predictive of coronary heart disease in a developing community: principal results of the St James Survey, Trinidad | Trinidad and Tobago | Community | Cohort Study | Afro-Caribbean, Indian, European | Incidence of CHD |  | Ethnicity, Age, Sex | No reported limitations. |
| Sarti, C  2000 | International Trends in Mortality From Stroke, 1968 to 1994 | WHO database including data from Trinidad and Tobago | Community | Cross sectional | Age range 35-84yrs | Mortality from Stroke |  | Age, Sex | Routine mortality data collected from so many countries are not reliable enough to allow subtype-specific analyses. Lack of information on case fatality, which requires incidence data to be calculated. Without case-fatality and subtype analyses, we can only speculate on the reasons for the changes in trends. |
| Smeeton, N 2011 | A comparison of outcome for stroke patients in Barbados and South London | United Kingdom | Community (stroke registry) | Cross sectional | Barbados Stroke Registry (BROS) Afro-Caribbean n= 924, Caucasian n= 43  South London Stroke Registry (SLSR) Afro-Caribbean n= 121, Caucasian n= 736  Mean age: BROS 71.3(15.4)  SLSR 69.7 (15.1) | Mortality | Institutional care | Geographic location, Ethnicity | Strength of this study is that it uses detailed information collected through population-based stroke registers, both of which have been established for several years. Although the sample size was relatively large, it was not possible to analyze the black Caribbeans as a separate group due to the lack of statistical power. |
| Summerell, J  1968 | Autopsy data on heart disease in Jamaica | Jamaica | Hospital | Case series | Black Caribbean  Men n= 855  Women n= 656  Age range 17- 80 yrs.  Lower SES group | Mortality from Heart disease |  | Age, Sex | No reported limitations. |
| UKPDS-Research Group  1998 | The incidence of myocardial infarction in white, South Asian, and Afro-Caribbean patients with type 2 diabetes (U.K. Prospective Diabetes Study 32) | United Kingdom | Hospital | Cohort study | White: men n= 2367, women n= 1734  South Asian: men n= 336, women n= 154  Afro-Caribbean: men n= 217, women n= 166  Mean age: Overall 52 (25-65) yrs.  Whites 52.2 yrs., South Asians 47.0yrs, Afro-Caribbean 52.3 yrs. | Incidence of fata and no-fatal MI |  | Socio-economic status, Sex, Age | No reported limitations. |
| Wang, Y  2013 | Trends and Survival Between Ethnic Groups After Stroke: The South London Stroke Register | United Kingdom | Community (stroke registry) | Longitudinal study | Ethnicity: White n= 3005, Afro-Caribbean n= 536,Black African n=296, Other 375  Gender: 50.3% men  Mean Age range: Afro-Caribbean 66.6, Black African 56.7, Whites 72.8 | Prevalence of stroke risk factors at baseline; post stroke survival |  | Ethnicity | Case ascertainment was an issue. Under-ascertainment of stroke cases among blacks. The possibility of differential case ascertainment in different ethnic groups may not be completely ignored and may influence the interpretation of the survival data. |
| Wild, S  1997 | Cross sectional analysis of mortality by country of birth in England and Wales, 1970-92 | United Kingdom | Community | Cross sectional | Population from the South London Stroke Registry  Age range: 20-69 yrs. | Mortality from Ischaemic heart disease (IHD) and stroke |  | Age, Sex, Ethnicity | No reported limitations.  Analysis used standardized mortality ratios for comparison. |
| Zaninotto , P 2007 | Prevalence of cardiovascular risk factors among ethnic groups: results from the Health Surveys for England | United Kingdom | Community | Cross sectional | Ethnic groups: Afro-Caribbean, South Asians, Chinese, Irish and UK Whites. | Prevalence of CHD |  | Ethnicity, Sex | Given the cross-sectional nature of this study, the direction of the relationship between variables cannot be assessed. Another possible limitation is that the individual response rates for the general populations were not as high as the response rates for the minority ethnic groups. |
